# The triune of intestinal microbiome, genetics and inflammatory status and its impact on the healing of lower gastrointestinal anastomoses

**DOI:** 10.1111/febs.14346

**Published:** 2017-12-22

**Authors:** Jou A. Lee, Timothy J. A. Chico, Stephen A. Renshaw

**Affiliations:** ^1^ Department of Infection Immunity and Cardiovascular Disease The Bateson Centre University of Sheffield UK

**Keywords:** anastomotic leak, colorectal surgery, microbiota, polymorphism, surgical inflammation

## Abstract

Gastrointestinal resections are a common operation and most involve an anastomosis to rejoin the ends of the remaining bowel to restore gastrointestinal (GIT) continuity. While most joins heal uneventfully, in up to 26% of patients healing fails and an anastomotic leak (AL) develops. Despite advances in surgical technology and techniques, the rate of anastomotic leaks has not decreased over the last few decades raising the possibility that perhaps we do not yet fully understand the phenomenon of AL and are thus ill‐equipped to prevent it. As in all complex conditions, it is necessary to isolate each different aspect of disease for interrogation of its specific role, but, as we hope to demonstrate in this article, it is a dangerous oversimplification to consider any single aspect as the full answer to the problem. Instead, consideration of important individual observations in parallel could illuminate the way forward towards a possibly simple solution amidst the complexity. This article details three aspects that we believe intertwine, and therefore should be considered together in wound healing within the GIT during postsurgical recovery: the microbiome, the host genetic make‐up and their relationship to the perioperative inflammatory status. Each of these, alone or in combination, has been linked with various states of health and disease, and in combining these three aspects in the case of postoperative recovery from bowel resection, we may be nearer an answer to preventing anastomotic leaks than might have been thought just a few years ago.

**Abbreviations**
AL: anastomotic leak
COX: cyclooxygenase
CYP: cytochrome p450
Gf: germ‐free
GIT: gastrointestinal tract
HBOT: hyperbaric oxygen therapy
HIF: hypoxia‐inducible factor
ICG: indocyanine green
IFN: interferon
IL: interleukin
LPS: bacterial lipopolysaccharide
LOX: lipoxygenases
NSAIDs: nonsteroidal anti‐inflammatory drugs
PG: prostaglandin
PHD: prolyl hydroxylase domain
PUFA: polyunsaturated fatty acid
SNP: single nucleotide polymorphism
TNF: tumour necrosis factor

## Introduction

Treatments for haemorrhoids have been described since the 12th and 13th century BC, but direct surgical intervention in other parts of the bowel was actively avoided in ancient times, being performed infrequently even in acute injuries, and almost always ending dismally 1. It was only in 1783 that Dubois performed the first recorded operation on uninjured bowel in the form of a colostomy for anorectal malformation in a baby, who survived only 10 days 2. Another 40 years would pass before the first successful segmental colonic excision was performed in 1823, but this was followed soon after by a successful anorectal excision in 1826 3. Since then, the numbers of lower gastrointestinal tract (GIT) operations have grown exponentially, alongside refinements in operative technique and technology. In the UK alone, between 2005 and 2014, traditional open surgery for colonic resections persisted at approximately 25 000 per annum, yet in addition, laparoscopic resections increased sevenfold to over 7000 cases in 2014 4. The rising number of lower GIT resections comes as no surprise due to increased public awareness of symptoms of bowel pathology, in combination with screening programmes performed in an ageing population. Reassuringly, improvements in survival have been substantial compared to that first foray into GIT surgery in the 18th century. But one complication continues to haunt the surgeon, and indeed, the surviving patient: the anastomotic leak (AL).

Many resections now involve an anastomosis to rejoin the ends of the remaining bowel, thus restoring GIT continuity. While most heal uneventfully, in up to 26% of patients 5 healing fails and an anastomotic leak (AL) develops despite the best technical efforts. When an anastomotic leak occurs, intestinal contents leak into the peritoneal cavity. Intestinal contents contain multiple microorganisms and either digestive enzymes or frank faeces, depending on the location of the anastomosis within the GIT. This can result in a range of complications such as abscesses, erosion of surrounding tissues causing abnormal connections (fistulae), generalised infection of the peritoneum (peritonitis) with an accompanying systemic inflammatory response, and even multiorgan failure and death. Because of the serious consequences of failure of healing, decades have been dedicated to optimising operative techniques such as accurate apposition of tissues with submucosal suturing to evert the mucosal layer for a leak‐free join, careful preservation of the vascular arcades to the ends of the anastomosis with visible perfusion and maintenance of a sterile operating field. When these failed to eliminate leaks, further fine‐tuning extended to the perioperative period with careful control of blood pressure and albumin levels, in addition to advances in surgical technology resulting in staplers, fibrin glue and nickel‐titanium compression ring anastomotic techniques 6. But none of these have proven infallible. For decades, surgeons have puzzled over this conundrum and searched for additional surgical or patient risk factors, such as smoking, male sex, anastomoses close to the anal verge, high arterial ligation or preoperative radiotherapy 7, 8, 9, 10. Many risk stratification strategies have emerged but again, none consistently predict 11, 12, 13 or explain this often‐catastrophic complication. Indeed, despite increasing sophistication in surgical and anaesthetic techniques, the rate of anastomotic leaks has not decreased over the last few decades 14. This raises the possibility that perhaps we do not yet fully understand this phenomenon of ALs and are thus ill‐equipped to prevent it.

**Figure 1:**
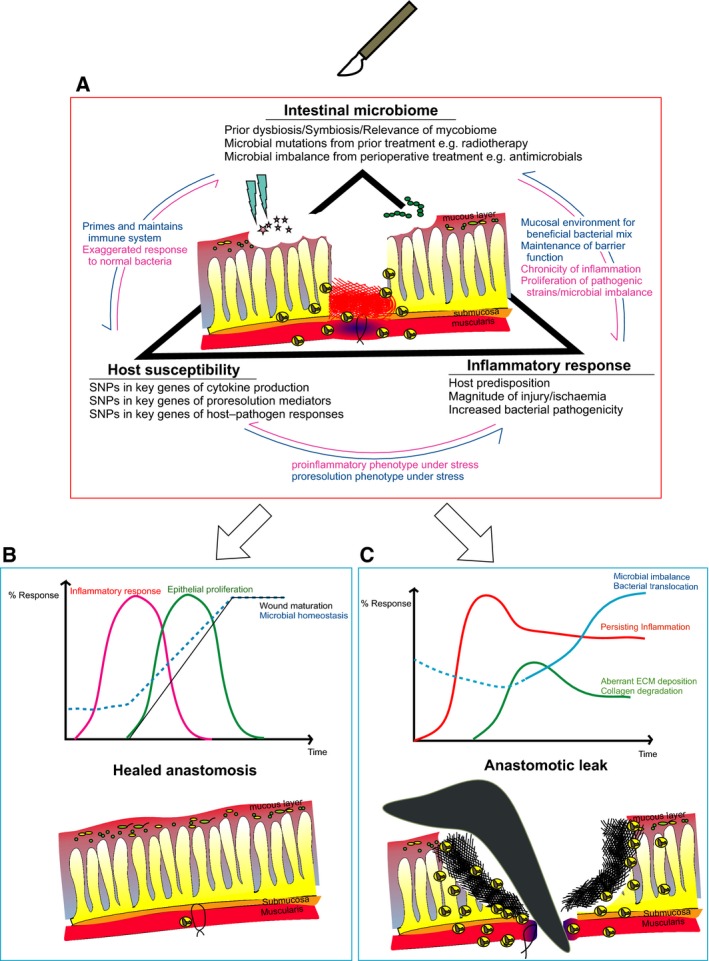
Surgical resection and anastomosis of the bowel (A) where wound healing is influenced by three interdependent factors: host genetic susceptibility, inflammatory response and the existing intestinal microbiome. The interaction of these three factors leads either to (B) healing of anastomosis with epithelial restitution, resolved inflammatory responses, restored mucous barriers and microbial homeostasis; or to (C) failed healing leading to an anastomotic breakdown, leakage of intestinal contents, bacterial translocation and systemic sequela. Graphs adapted from Clarke, RAF (1996) *The molecular and cellular biology of wound repair*.

So what do we know so far about the reasons anastomoses leak, and where might the future lie for research in this area? As in all complex conditions, it is necessary to isolate different aspects of disease for interrogation of its specific role, but, as we hope to demonstrate in this article, it is a dangerous oversimplification to consider any single aspect as the full answer to the problem. Instead, consideration of important individual observations in parallel could illuminate the way forward towards a possibly simple solution amidst the complexity. Literature directly related to molecular aspects of anastomotic healing is less extensive than would have been ideal, perhaps because there are technical challenges in creating an intestinal wound and subsequently directly observing healing in an internal organ in a clinically relevant manner. Also, it is only recently that the unique aspects of wound healing in the GIT distinct from that of other epithelial surfaces such as the skin or even lung are being recognised. This viewpoint article details three aspects that we believe intertwine, and therefore should be considered together in wound healing within the GIT, during postsurgical recovery: the microbiome, the host genetic make‐up and their relationship to the perioperative inflammatory status (Fig. 1).

## The role of inflammation

One of the defining observations in early medicine was that inflammation accompanies injury. First described by Celsus in 1AD 15, the cardinal features of inflammation, to different magnitudes, inevitably follow invasive surgical procedures. Many of the basic tenets of good surgical practice such as meticulously gentle tissue handling, preserving a good blood supply, debridement of nonviable tissue and asepsis, may have their molecular basis in minimising the magnitude of inflammation that occurs. While there is certainly no place for gross departure from these principles, how important are they in preventing AL and how might they relate to cellular and molecular aspects of inflammation?

Many pharmacological and mechanical aspects have been reviewed 16 over the years and a selected few are summarised in Table 1. But interpretation of these experimental results is more complex than would seem at first glance. Let us examine, as an example, the importance of a good blood supply to the ends of the bowel forming the anastomosis. It almost defies common sense that anything could be more important than this. And yet experimental evidence is conflicting. Early experiments in dogs with intentional vascular occlusion causing 5 cm of visible devascularisation of the anastomotic region showed that in the sterile bowel, maintained by intraluminal antibiotic treatment, anastomoses healed despite this ischaemia 17. More recent examples in rodents with segmental colonic ischaemia at the sites of anastomoses suggested a lack of difference in levels of tissue hypoxia between healed or nonhealed anastomoses 18. However, in other studies using more severe arterial occlusion, positive effects with hyperbaric oxygen therapy (HBOT) have been demonstrated in healing, and negative effects with systemic hypoxia 19, 20. In hypoxia, it appears logical that decreasing the partial pressure of available atmospheric oxygen would subsequently decrease the haemoglobin saturation, and thus push oxygen delivery below the threshold for wound healing in a situation of tenuous vasculature. Conversely, in hyperbaric oxygen therapy (HBOT), where 100% oxygen is given at higher than atmospheric pressure, not only are red blood cells fully saturated but also blood can become hyperoxygenated by plasma saturation as well, leading to possibly adequate oxygen delivery despite fewer vascular connections.

**Table 1:** Exemplar interventions for prevention of ALs and their relationship to the inflammatory process. This list includes some of the options that have been investigated, how they might ultimately impact upon the inflammatory pathways and their often divergent conclusions

Intervention investigated	Relationship to the inflammatory process	Author/Year	Conclusion
Ischaemia prevention	PHD/HIF pathways (see text)	Cohn & Rives 1956 17	Ischaemia did not cause AL in a sterile bowel in dogs
		Shaksheer *et al*. 2016 117	No difference in tissue hypoxia levels in mice
		Schouten *et al*. 2014 22	No differences in microvessel density in human histology specimens
		Pommergaard *et al*. 2015 118	Impaired blood supply impairs healing in mice
		Attard *et al*. 2005^19^	Systemic hypoxia impairs healing in rats
		Boersema *et al*. 2016 20	HBOT improves anastomotic healing in rats
Electromagnetic field therapy (EMF)	Electromagnetic field therapy can downregulate inflammatory cytokines (IL6, IL1 COX2) and upregulate IL10 119, 120	Mente *et al*. 1996 121	Improved mechanical strength and hydroxyproline content with EMF in mice
		Nursal *et al*. 2001 122	No difference in mice
Amniotic membrane	Amniotic membranes have integral immunomodulatory properties to avoid rejection of foetus	Barski *et al*. 2016 123	Increased inflammation and adhesion, does not prevent AL in mice
		Moslemi *et al*. 2016 124	Prevents AL and reduced adhesions in rats
Fibrin glue/sealants	Mechanical barrier reducing perianastomotic Inflammation	Pantelis *et al*. 2010 125	Positive effect with fibrin glue in healing in mice
		Senol *et al*. 2012 126	Improved hydroxyproline content with fibrin glue in rats
		Nordentoft *et al*. 2015 127	A review showing lack of effect of fibrin glue
		Slieker *et al*. 2013 128	No effect of six different sealants in mice
Sildenafil	Decreased neutrophil infiltration/decreased cytokine (see text)	Cakir *et al*. 2015 129	Improved collagen maturity in rats
		Irkorucu *et al*. 2009 130	No effect on anastomotic integrity in rats

But even if we suppose that perhaps the submucosal plexus of the intestine is more robust in animals and that experimental models do not adequately recapitulate human physiology, clinical reports also conflict. While reduced microperfusion of the rectal stump has been linked to ALs as might be expected 21, a separate histological study of microvessel density revealed no difference between AL and healed specimens 22. This confusion surrounding the hierarchical importance of blood supply has not slowed efforts to improve intraoperative visualisation of perfusion, with innovations such as scanning laser Doppler flowmetry 23 and fluorescent indocyanine green (ICG) being introduced in recent years. But again, for example with ICG, there is still a lack of randomised controlled trials evidencing a significantly reduced AL rate 24. Although nonrandomised studies have indicated no leaks in the anastomoses that were immediately revised based on intraoperative ICG fluorescence, it is unknown if these would ultimately have leaked without revision 25.

Perhaps some of the difficulties in obtaining a clear‐cut conclusion are because the hypoxia‐inflammation axis is more complex within the bowel, compared to other mucosal surfaces like the lung. The bowel is unique in that highly vascularised intestinal structures are in proximity to a physiologically hypoxic lumen especially within the lower GIT 26. Studies in dogs suggest the existence of a counter current flow within small intestinal villi which allows this permissive hypoxia 27, although there is absence of evidence for this in other mammals such as the rat 28, and the picture is complicated by fluctuations in blood flow from postprandial hyperaemia. But within the colon, where postprandial hyperaemia has not been demonstrated, the steep hypoxic gradient appears to be maintained by subsets of facultative anaerobes residing close to the mucosa 29. In line with this gradient, the GIT is well adapted to tolerate hypoxia and intestinal epithelial cells express the three hypoxia‐inducible factor (HIF) α subunits and the constitutive HIF‐1β subunit that completes the heterodimer, as well as their inhibitors, the three prolyl hydroxylases (PHDs) and factor‐inhibiting HIF1 (FIH1) 30. Under basal conditions of physiologic hypoxia, HIF‐1α is stabilised and, as a transcription factor, serves to perform enhanced barrier integrity functions in the gut by increasing expression of a multitude of genes such as those coding for mucin 31 and antibacterial peptide production 32. As oxygen becomes plentiful, PHDs hydroxylate the inducible α subunit and target it for degradation. So central is the role of HIF1 in maintenance of mucosal homeostasis that inhibitors of PHDs are being trialled clinically as treatment for the chronic inflammatory states of Crohn's disease and ulcerative colitis 33, 34.

It is well established that the early stages of inflammation involve an influx of innate immune cells, neutrophils followed by macrophages, which patrol the inflamed area removing debris and invading pathogens. Neutrophils are primarily glycolytic 35 and therefore able to function without detriment in environments of low oxygen tension. However, neutrophils also increase hypoxia in inflammatory environments as they consume many more times the amount of oxygen than other cells, when generating an antimicrobial oxidative burst 36. While it might be imagined that hyperbaric oxygen therapy (HBOT) in the animal experiments above acts solely by reversing this hypoxia, the picture is more complex. HBOT in fact also acts in an anti‐inflammatory manner within the intestine, and has been shown to directly block interleukin‐1β (IL‐1β) and increase HIF‐1α mRNA 37. This is possibly through stabilisation of HIF‐1α via formation of oxygen radicals, but exact mechanisms are as yet unclear 37. Other studies of HBOT in the acute inflammatory process also demonstrate a downregulation of cyclooxygenase‐2 (COX2) mRNA 38, highlighting the anti‐inflammatory role of this therapy. A similar example of the complexity of mechanism of action is with sildenafil, a vasodilator beneficial in anastomotic healing 39, but which also has independent anti‐inflammatory properties, and decreasing TNF‐α and interleukin‐6 (IL‐6) in human studies 40. The effect of inflammation is also important in other areas that may indirectly impact upon healing. One example is that of postoperative ileus, where normal GIT motility fails to return in a timely manner. The sympathetic neural response to intrusion of the peritoneal cavity and handling of bowel causes a reactive hypomotility of the GIT, which usually fades quickly upon abdominal closure, but prolonged ileus can occur, has been shown to be associated with increased IL‐6 and leucocyte infiltration of the bowel 41, 42, and has a positive correlation with rates of AL 43. Here again evidence shows that inhibiting the inflammatory axis via COX‐2 improves ileus 44, 45. Some clinical studies comparing laparoscopic with open surgery suggest a relationship between decreased inflammation, as evidenced by a lower CRP level postlaparoscopy, with lower ileus and AL rates 46, but caution needs to be exercised in interpreting these results as correlation does not always imply causation.

Indeed, eliminating inflammation *per se* does not seem to be a panacea because, paradoxically, anti‐inflammatory drugs have met with unexpected failure in preventing ALs. Although prescribed mainly for analgesia rather than reduction of inflammation, NSAIDs are associated instead with an increase in AL 47, 48. This may be because, while COX‐1 is constitutively expressed by intestinal epithelial cells, COX‐2 expression is initiated by the presence of inflammatory mediators. Recent studies in mice have identified COX‐2‐dependent production of PGE2 as essential for neovascularisation and subsequent intestinal healing, with disruption of this pathway increasing ALs 49. Other inflammatory mediators such as IL‐6 have also been shown to be essential during the initial injury phase to promote epithelial reconstitution within the intestine 50. Certainly sound surgical technique cannot be sacrificed for molecular nuances, but what on the surface may appear to be due merely to sensible surgical technique may be much more complex than previously thought.

Interestingly, in the experiments above, administration of PGE2 alone did not fully rescue the COX‐2 knockout AL phenotype 49. As the authors suggest, this could be due to dosage, as in mice there appears to be a high sensitivity to the loss of even one functional allele of the parent gene 51, or perhaps due to the lack of other eicosanoids such as PGI2 or specialised proresolving mediators such as lipoxins and resolvins. But there is also evidence that TLR4 and MyD88 signalling pathways, central in host–pathogen interactions, are required for optimal tissue repair in intestinal wounds 52. All these reinforce the suggestion that the pathways of inflammation and healing are complex and closely intertwined, particularly in the context of the GIT where there is a close presence of a multitude of microbes, such that it becomes difficult to tease out the dominant (if any) pathway leading to a beneficial or detrimental effect. In the example of HBOT, not only are anti‐inflammatory pathways activated but the increased pressure of oxygen also perturbs the hypoxic gradient of the intestine and alters the composition of up to 29 species of bacteria within the rodent intestine 29. This has the potential for wide‐ranging consequences, as the inflammatory responses are also primed by the intestinal microbiome. A study comparing the inflammatory responses of germ‐free (Gf) and conventional mice to bacterial lipopolysaccharides and ischaemic injury showed that Gf mice had a significantly increased lipoxin‐induced production of IL‐10 and a dampened inflammatory response 53. Similarly, over half a century ago, postoperative ileus itself was shown to improve with administration of antibiotics 54. So could the traditional thinking of complete eradication of microbiota to minimise surgical site infections also ameliorate ALs?

## The role of the intestinal microbiome

The field of microbiomics has rapidly expanded in recent years with sequencing techniques that allow identification of nonculturable bacteria 55. The identification of this vast community of microbiota quickly led to demonstration of the consequences of intestinal microbiota in diseases once thought to be far removed and distinct from the gut, such as hypertension 56 and Parkinson's Disease 57. But as interactions between distant systems are increasingly uncovered, interest in the relevance of gut microbiota closer to home, within the GIT itself, has also had a revival with excellent reviews published suggesting an important role for microbiota in wound healing in surgery and also in GIT cancers 58, 59, 60, 61. Intestinal antisepsis is not a new concept in GIT surgery particularly in the lower GIT which harbours the highest concentration of bacteria within the human body under conditions of health. Antibiotics were used in colorectal surgery as early as 1938, and since the work of Poth, Cohn and Cohen 59, 62, it is now widely accepted that intestinal microbes play important roles in ALs.

We know that although the core milieu of microbiota within the GIT persists stably in an individual 63, it is still changeable and alters over time with age 64, and within hours to dietary and lifestyle changes such as exercise, smoking 65, 66, 67 and indeed surgery. Experiments in rats demonstrated a 500‐fold increase in *Enterococcus* and 200‐fold increase in *Eschericaea*, in conjunction with a 20‐fold decrease in *Ruminococcacea, Clostridia* and *Prevotellaceae* 68 following a surgical procedure. A similar change was demonstrated in human patients undergoing a colectomy, again with an increase in *Enterobacteriacaea, Enterococcus, Staphylococcus,* and *Pseudomonas* (an aerobe), and a decrease in obligate anaerobes like *Bifidobacterium* 69.

These changes are of particular interest as *Enterococcus faecalis* is potentially directly responsible for ALs by degrading collagen through expression of gelatinase E 70. Emerging evidence also suggests that prior radiotherapy, as is commonly used for some rectal cancers, may cause mutations in bacteria such as *Pseudomonas* that increase bacterial virulence. In a rat model of rectal surgery, pretreatment with radiotherapy in the presence of *Pseudomonas* significantly increased ALs due to a mutation increasing pyocyanin and collagenase activity 71. This was in clear contrast to the complete lack of ALs in rats who underwent radiotherapy and an anastomosis but were free of *Pseudomonas*. These appear to be compelling pieces of evidence that eradication of microbiota may hold the key to ameliorating ALs; so much so that a recent summary of clinical trials to date 59 concluded that routine bowel preparation with nonabsorbable antibiotics should be recommended. But could it be that the resultant significant changes in intestinal microbiota, as would be the case even for narrow spectrum antibiotics, may cause more harm than good both in the short and long term? It is indisputable that prolonged and repeated courses of antibiotics and polypharmacy 72, 73 influence the long‐term diversity of the microbiome, but even a single pulse of broad‐spectrum antibiotics in mice appear to cause long‐term microbiome changes 74 which ultimately have the potential to negatively impact later physical health (such as growth, diabetes and obesity), mental health 75, 76, 77 and even mortality 72, 78.

It is in fact quite impossible to eradicate intestinal microbiota in its entirety, because it would be necessary to ensure that the chosen antibiotic would be able to reach and eradicate both autochthonous (resident) microbes and not just allochthonous (luminal transient) microbes throughout the length of the GIT. Even then, such an action would be undesirable as the risk remains that dormant spores such *Clostridium difficile* might reactivate causing serious disease 79. But quite apart from that, evidence is accumulating that the presence of the microbiome may be beneficial, and even necessary, for optimal wound healing, in addition to its other whole organism effects. *In vitro* studies have shown that *Akkermansia muciniphila* and *Bacteroides fragilis* significantly improved gut epithelial integrity, and furthermore survive normoxic conditions despite being anaerobes 80, an important consideration as exposure to environmental oxygen during a surgical resection temporarily increases oxygen tension within the lower GIT and diminishes obligate anaerobes. These bacterial effects have been replicated *in vivo* in mice with encouraging results showing accelerated mucosal re‐epithelialisation 81, 82. Conversely, a lag in skin wound healing in Gf guinea pigs has been noted from experiments in the 1960s 83, with similar findings of a lower tensile strength of intestinal anastomoses in Gf mice 84, 85.

But in addition to the positive effects on wound healing, it is increasingly recognised that intestinal microbiota are directly implicated in the proper development of the immune system. The presence of intestinal commensals is vital in the development of gut‐associated lymphoid tissues, secretory IgA and Th17 cells, a subset of T‐helper cells important in inflammation 86, 87. But, as mentioned previously, the importance of microbiota does not end in development. Throughout life, the microbiome continues to exert a tonic effect on the inflammatory response. Supplementing *Bifidobacteria* to postoperative patients decreased proinflammatory cytokines and enhanced recovery 88, and the gut commensal *Faecalibacterium prausnitzii* orchestrates anti‐inflammatory effects by inducing secretion of IL‐10 and reducing IL‐12 and IFNƔ 89. Even a single factor, Polysaccharide A from *Bacteroides fragilis*, is able to induce production of IL‐10 and confer protection from experimental colitis in animal models 90.

Furthermore, in all discussions of antimicrobials, it is of vital importance to remember that it is not only bacteria that make up the intestinal biomes. Fungal, archaea and viral entities also coexist 91, 92, and their roles have yet to be fully explored in the pathogenesis of a multitude of conditions. It was only 2 years ago that a study demonstrated an increased fungal load in the chronically inflamed intestines of Crohn's patients 93 even though fungi have been identified in the GIT for over a century 94. Over the past decade, experiments in rodents have identified a rich mycobiome that exists alongside the microbiome, and which similarly influences health and disease. Mice lacking the innate immune receptor Dectin‐1, which is also a fungal signalling receptor, are highly susceptible to severe colitis, paralleling observations in humans 95, suggesting that fungal presence may add some benefit to the overall homeostasis of the GIT.

Although yet to be demonstrated specifically in anastomotic wounds, evidence also exists that fungal pathogenicity increases with antibacterial therapy, and are implicated in necrotic or nonhealing wounds 96. Interestingly, within the intact gut of the worm, *Candida albicans* and *Enterococcus faecalis* show a symbiotic relationship, concurrently reducing the pathogenicity of the other. In the absence of *E. faecalis*,* C. albicans* showed an increased hyphal morphogenesis, a key virulence factor 97. This type of relationship is not limited to the GIT. Even in the skin, cocolonisation of pathogenic *Staphylococcus aureus* with the commensal, *Corynebacterium striatum,* caused a downregulation of *Staphylococcal* haemolysin activity and a shift towards commensalism 98. And in the urinary tract, escherichelin production by *Enterobacteriae* cause an inhibition of growth of *Pseudomonas* by competitive inhibition of iron transport 99. These examples should serve to promote caution in advocating blanket antimicrobials for the removal of any one species of bacteria shown to be responsible for ALs within the highly regulated environment of an animal facility, as the results may not be quite as straightforward in real‐world human cases.

While evidence is mounting that the composition of intestinal microbiota is vital in determining the final outcome of an anastomosis and that it is eminently malleable by exogenous supply or by dietary and antimicrobial manipulations, it would be rather difficult to ensure the ideal mixture of microbes for perfect wound healing be present through standardised prescriptions. This is especially so as interindividual variation is substantial and it is not yet fully understood how an individual selects or maintains the composition of their personal microbiome. This continues to be an area of active research, but already it is emerging that genetic variation is one of the key factors in controlling inflammatory responses to the same microbial stimulus 100 and possibly the resulting dysbiosis associated with various illnesses.

## The role of the host genetic make‐up

The inflammatory response to injury and infection has to be carefully controlled to avoid disease states. Classical eicosanoids, docosanoids, as well as the newer bioactive lipid mediators play important roles in this process. But while knowledge is increasing in these areas, the pathways remain highly complex, having a dynamic expression that is dependent on many factors such as time, tissue specificity and cell–cell interactions 101. This makes it challenging to isolate the effects of inhibiting a single pathway, as knock‐on effects on other pathways are often present and not always predictable particularly in the early stages. The lipid signalling and metabolism pathway starts with a supply of arachidonic acid liberated by the phospholipase A2 enzyme, and ω‐6 and ω‐3 polyunsaturated fatty acids (PUFAs). These are then further modified by cyclooxygenase (COX), lipoxygenase (LOX) or cytochrome p450 (CYP) enzymes 101 into many different end products. Over the past decade, the importance of these lipid pathways in human disease has been investigated in some detail 102. Although the primary end‐point in many of these studies was cancer, they remain relevant because of implications for the role of inflammation, which has been shown to have both a cell‐autonomous and non cell‐autonomous function in promotion of carcinogenesis 103. Epidemiological studies show that up to 20% of cancers are the direct result of chronic inflammation 104, and over the past century, tumour initiation, maintenance and progression has been proven to have clear molecular similarities with the nonhealing wound 105, 106, 107, and a strong association with the prevailing microbial landscape 108. Therefore, polymorphisms in pathways found to be important in cancer prevention or development are also likely to be important in prevention of or predisposition to ALs.

Humans are unable to convert ω‐6 PUFAs into ω‐3 PUFAs and therefore the availability of each in the diet corresponds to its abundance within the body thus facilitating epidemiological studies. Cohort studies with manipulation of the exogenous supply of each (ω‐6 in excess being proinflammatory and ω‐3 anti‐inflammatory) have been performed, which were then correlated to cancer risk. Results of these early studies have been contradictory and overall failed to definitively associate a reduction in sporadic forms of cancer with increased ω‐3 PUFA consumption. These equivocal results can partly be explained by newly discovered single nucleotide polymorphisms (SNPs) within the COX genes, such that only those with a proinflammatory COX1 variant derived benefit from ω‐3 supplementation 109. The converse is also true, albeit shown in a distinct Chinese population, with proinflammatory COX2 polymorphisms conferring elevated risk when combined with increasing ω‐6 PUFA consumption 110.

Multiple experimental and clinical studies have investigated links between various nongenetic aspects thought to be important in anastomotic healing and in many cases results have not been immediately clear, or have even been conflicting. Given similar difficulties described above with establishing cause‐and‐effect in PUFAs and cancer, it is likely that subtle genetic differences in these pathways also underlie susceptibility to ALs. Some progress has been made to identify genetic differences in postoperative complications such as pneumonia, once thought to be mainly due to a mechanical effect of reduced inspiratory depth following a laparotomy, but where an IL‐10 polymorphism has been shown to be an independent risk factor 111. Various cytokine polymorphisms have also been implicated as risk factors in the development of severe sepsis 112, but strong links specifically to ALs is still in its infancy. We speculate that future work would be likely to show the central importance of these pathways of inflammation in ALs.

For example, while a low anastomosis has been identified as a risk factor for ALs 7, 8, 9, 10, the molecular explanation for this has been somewhat elusive. But perhaps the answer lies in the genetic predisposition of the patient. A SNP in PTGS2 (−765G>C; rs20417) resulting in decreased COX2 expression has been strongly associated with the risk of developing rectal cancer but not colon cancer 113. And as mentioned previously, COX2 plays a vital early role in promoting the angiogenesis important for anastomotic healing. This could be an explanation for the increased leak rate in rectal cancer surgery compared to colonic cancer surgery. Similarly, SNPs in the LOX pathways, for example that of lipoxygenase‐15 (ALOX15 1351G>A), have been positively associated with the risk of rectal, and not colon, cancer 114. As the products of ALOX15 such as the lipid mediator lipoxin A4 are also mediators of inflammation resolution, this hints at yet another subtle but important role of host genetics on inflammation and an increased risk of ALs. Thus far, a single study has identified a link between homozygosity in the PTGS2 SNP conferring rectal cancer risk and an increased risk of ALs, even though a distinction between colon or rectal resections was not made 49. But the complexity of this area of research is exemplified by the knowledge that the prevalence of this polymorphism is only 3% 115, it is not the only polymorphism of significance, and differences may be present in accordance with ethnicity 116.

## Conclusion

It is an exciting time for researchers in this field as significant progress is being made in each of the three fundamental areas that ultimately converge for bowel healing. It is clear that there still remains work to be done to unite these areas in our understanding of the molecular underpinnings of ALs. While it is still early days for investigation of genetic polymorphisms in postoperative complications, it is an area likely to be extremely complex due to the vast possibilities of candidate genes if assessed independently. But it seems likely that, for the future, the most productive translational impact will necessarily be found in integrating all three aspects to drive the field forward with personalised therapeutic options based upon the genetic predisposition of the patient. The complexity of GIT homeostasis dictates that, while it is certainly helpful to dissect molecular targets and pathways by investigating isolated aspects, it is vital that all three fundamental areas be considered together in order to make a meaningful step towards translational therapeutic strategies to prevent the dreaded complication of ALs once and for all.
